# Dynamin-dependent NMDAR endocytosis during LTD and its dependence on synaptic state

**DOI:** 10.1186/1471-2202-6-48

**Published:** 2005-07-22

**Authors:** Johanna M Montgomery, Joel C Selcher, Jesse E Hanson, Daniel V Madison

**Affiliations:** 1 Department of Molecular and Cellular Physiology, Beckman Center for Molecular and Genetic Medicine, Stanford University School of Medicine Stanford, CA, 94305 USA; 2 Department of Physiology, Faculty of Medical and Health Sciences, University of Auckland, New Zealand

## Abstract

**Background:**

The N-methyl-D-aspartate (NMDA)-type glutamate receptor expressed at excitatory glutamatergic synapses is required for learning and memory and is critical for normal brain function. At a cellular level, this receptor plays a pivotal role in triggering and controlling synaptic plasticity. While it has been long recognized that this receptor plays a regulatory role, it was considered by many to be itself immune to synaptic activity-induced plasticity. More recently, we and others have shown that NMDA receptor-mediated synaptic responses can be subject to activity-dependent depression.

**Results:**

Here we show that depression of synaptic transmission mediated by NMDA receptors displays a state-dependence in its plasticity; NMDA receptors are resistant to activity-induced changes at silent and recently-silent synapses. Once synapses transition to the active state however, NMDA receptors become fully 'plastic'. This state-dependence is identical to that shown by the α-amino-3-hydroxy-5-methylisoxazole-4-propionic acid (AMPA) receptor. Furthermore, the down-regulation of NMDAR-mediated responses during synaptic depression is prevented by disruption of dynamin-dependent endocytosis.

**Conclusion:**

NMDA receptor-mediated synaptic responses are plastic in a state-dependent manner. Depending on the plasticity state in which a synapse currently resides, NMDA receptors will either be available or unavailable for down-regulation. The mechanism underlying the down-regulation of NMDA receptor-mediated synaptic responses is endocytosis of the NMDA receptor. Other potential mechanisms, such as receptor diffusion along the plane of the membrane, or changes in the activity of the channel are not supported. The mechanisms of AMPA receptor and NMDA receptor endocytosis appear to be tightly coupled, as both are either available or unavailable for endocytosis in the same synaptic states. Endocytosis of NMDA receptors would serve as a potent mechanism for metaplasticity. Such state-dependent regulation of NMDAR endocytosis will provide fundamental control over downstream NMDA receptor-dependent plasticity of neuronal circuitry.

## Background

The circuit and neuro-cellular mechanisms that underlie learning and memory have occupied the interest of scientists for many decades. The most compelling and widely accepted theories of learning and memory hold that memories are stored at synapses. More specifically, memories are formed and stored by persistent increases and/or decreases in the amplitude of postsynaptic potentials evoked during synaptic transmission across excitatory glutamatergic synapses. The most widely studied of these changes in synaptic efficacy are long-term potentiation (LTP) and long-term depression (LTD). LTP is the persistent increase in the amplitude of excitatory postsynaptic potentials (EPSPs) that follows high-intensity activation of a glutamatergic synapse. Conversely, LTD is the persistent decrease in EPSPs that follows low-intensity activation of a glutamatergic synapse. The mechanisms that trigger these changes in synaptic efficacy are relatively well-understood [1], but the mechanisms that express efficacy changes are less so.

In recent years, it has become clear that the trafficking of glutamate receptors into and out of the postsynaptic membrane plays a central role in the modulation of synaptic strength. Synapses can be potentiated or depressed in an activity-dependent manner through the postsynaptic insertion or internalization of the subtype of glutamate receptor known as the AMPAR (α-amino-3-hydroxy-5-methylisoxazole-4-propionic acid receptors) [2-4]. In most cases, this insertion and retrieval of AMPA receptors is triggered by calcium influx through another glutamate receptor subtype, the N-methyl-D-aspartate receptor (NMDAR). This has led to the concept that AMPARs are the receptors responsible for the expression of synaptic plasticity, while NMDARs are responsible for its control.

With regard to AMPAR-trafficking-mediated plasticity, increases and decreases in efficacy do not occur in a manner where the synapse simply slides up and down a continuum of strength [5]. Rather synapses change their efficacy by jumping between discrete mechanistic states such that, along with strength changes, come changes in the rules governing receptor trafficking [6]. We have previously identified five such synaptic plasticity states (active, silent, recently-silent, potentiated and depressed [6]. The plastic state in which a synapse resides determines its potential for undergoing further synaptic plasticity. Such states may form part of the underlying mechanism for metaplasticity [7]. Silent synapses, whose postsynaptic membranes contain NMDARs but no functional AMPARs, can be converted via an intermediate state (see below) to 'active' synapses, i.e. those containing both AMPARs and NMDARs, by AMPAR insertion into the postsynaptic membrane [2,3,8-10]. Active synapses can be potentiated by a high-intensity stimulus, or depressed by low-frequency stimulation (LFS) of presynaptic axons [6,11-13]. Prolonged LFS can even result in the silencing of a synaptic connection [6]. However, even when present in the synaptic membrane, AMPARs are not always available for down-regulation. For example, recently-unsilenced synapses (i.e. those into which AMPARs have recently been inserted) cannot undergo synaptic depression in response to LFS [6]. Thus, AMPAR regulation appears to be linked to the state of the synapse.

With regard to NMDARs, previous studies have suggested that, unlike the AMPAR, NMDARs are not subject to activity-dependent down-regulation during LTD [14,15], and data have shown that potentiation of NMDAR-mediated responses does not occur during LTP [10,16]. Recently however, it has been demonstrated that synaptic currents mediated by NMDARs can be regulated by synaptic activity or other factors, particularly in the negative direction [6,17-20]. During synaptic depression at active synapses, NMDAR-mediated responses are suppressed in an NMDAR-dependent manner [6,18,19]. This depression is accompanied by a decrease in postsynaptic NMDA sensitivity [6,19]. Evidence of NMDAR endocytosis following application of exogenous agonists has been shown in heterologous and neuronal systems [21-23], but it is not known whether this endocytic process can be induced by synaptic activity or whether it underlies activity-dependent synaptic plasticity. In addition, whether NMDARs, like the AMPARs, are subject to plastic state-dependent regulation is unknown. Because currents carried by the NMDAR control and trigger synaptic plasticity, a state-dependence of this receptor would determine the ability of the synapse to undergo further plasticity.

## Results

### Control of NMDARs at silent synapses

**Figure 1:**
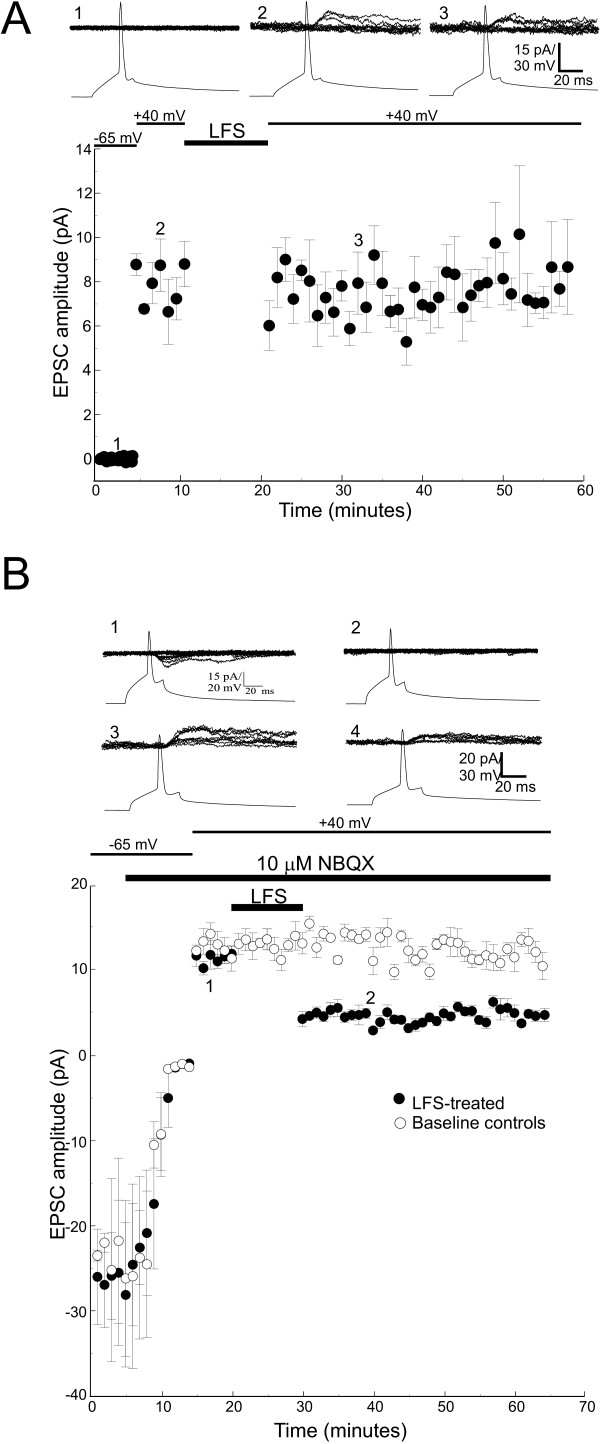
NMDAR-mediated responses are not depressed by low-frequency stimulation (LFS) at silent synapses in synaptically connected pairs of CA3 pyramidal neurons. **A. **Silent synaptic connections between pairs of CA3 pyramidal neurons [10]showed no synaptic responses when the postsynaptic cell was held at a negative potential (-65 mV). Depolarization of the postsynaptic cell to +40 mV revealed the NMDAR-mediated EPSC of these silent synapses. LFS did not cause any change in this NMDAR-only synaptic transmission (n = 7 pairs). Inset: Each inset shows consecutive postsynaptic traces overlaid, with one example presynaptic action potential. (1) 50 consecutive postsynaptic responses collected from the start of the paired recording, showing no AMPAR-mediated responses at -65 mV. (2). At +40 mV, NMDAR mediated responses are evident. (3) After performing LFS, NMDAR mediated responses are shown at +40 mV. For both (2) and (3), 10 consecutive traces are shown, before LFS (2), and ten minutes after LFS (3). **B. **The same LFS protocol applied to active synapses does depress the NMDAR-mediated EPSC (n = 6 pairs). After confirmation that a synaptic connection displayed AMPAR-mediated responses, NMDAR EPSCs were isolated by application of 10 μM NBQX. Inset shows ten consecutive postsynaptic traces collected at -65 mV before (1) and after (2) NBQX and +40 mV in NBQX, before (3) and 10 minutes after (4) LFS. An exemplar presynaptic action potential is shown in each case. Closed symbols: LFS-treated, open symbols, no LFS applied.

Silent synaptic connections contain only NMDARs; therefore how these receptors are regulated at these synapses will determine not only the regulation of the number of silent synapses, but also their future ability to contribute to synaptic potentiation. By recording from individual synaptically connected pairs of hippocampal pyramidal cells [10], synaptic plasticity can be studied in very small populations of synapses found in either a 'silent' or 'active' state. Recording from silent synapses between two neurons has enabled us to study NMDAR responses and their regulation in isolation. Synapse silence is demonstrated by the absence of an AMPAR-mediated component at a postsynaptic membrane potential of -65 mV in response to at least 50 consecutive presynaptic action potentials, beginning upon establishment of the postsynaptic cell whole-cell recording, followed by a demonstration that the connection has NMDAR-mediated EPSCs at depolarized potentials (Figure 1A). To determine whether NMDAR currents at silent synapses can be regulated by LTD-inducing protocols, we measured the amplitudes of these currents both before and after LFS. Unlike the suppression that occurs in active synapses [6] application of LFS (600 presynaptic action potentials at 1 Hz) had no effect on the amplitude of NMDAR responses in silent synapses (Figure 1A; average EPSC amplitudes were 7.9 ± 0.9 pA and 7.7 ± 1.1 pA (n = 7 pairs), before and after LFS respectively; p > 0.1). Similarly, LFS did not have any effect on the probability of NMDAR synaptic responses (average failure rates before and after LFS were 48.6 ± 5.7% and 49.8 ± 3.7% respectively; n = 7, p > 0.1) nor on the decay kinetics of the NMDAR EPSC (90 – 10% fall time 78.8 ± 18.9 and 79.7 ± 11.75 ms pre- and post-LFS respectively). This is in contrast to the behavior of NMDAR-mediated EPSCs in active synapses where LFS strongly suppressed the amplitude and increased the failure rates of NMDAR-mediated currents (Figure 1B). In these latter experiments, because of the presence of AMPAR-mediated responses at these active synapses, the NMDAR-mediated responses were isolated by bath application of the AMPAR antagonist NBQX (10 μM). On average, NMDAR EPSC amplitudes before and after LFS were 11.3 ± 0.6 pA and 4.8 ± 0.9 pA respectively (57.9% suppression, n= 6 pairs, p < 0.0001). This amplitude measurement is inclusive of synaptic failures. Since the failure rate of the NMDAR component of the EPSC increased from 10.3% to 36.6% (pre- and post-LFS respectively), the amplitude changes during LTD reported here will be an overestimate of the changes in "success" amplitude. Thus, the data on EPSC amplitude were analyzed an additional time with the failures excluded. In this analysis, the LFS-induced suppression of NMDAR-mediated responses was 41.2 +/- 10.0% (p < 0.01). This depression in EPSC amplitude was not accompanied by a change in NMDAR EPSC decay kinetics (97.33 ± 33.7 and 97.0 ± 38.3 ms pre- and post-LFS respectively).

### Control of NMDARs at recently unsilenced synapses

**Figure 2:**
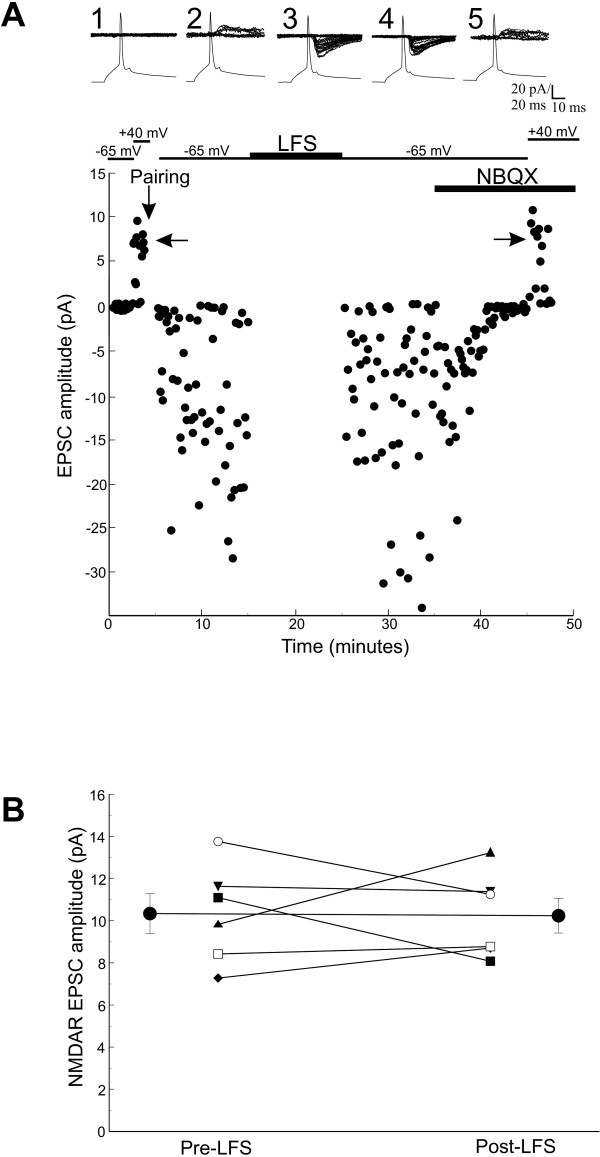
NMDAR-mediated responses are not depressed by LFS in recently unsilenced synapses. **A. **An individual experiment showing a silent synaptic connection with no AMPAR-mediated transmission at -65 mV. Depolarization of the postsynaptic cell to +40 mV revealed the NMDAR-mediated responses (left arrow). Following the induction of LTP by pairing (downwards arrow; [6]), AMPAR-mediated responses appeared (at -65 mV). LFS was performed 10 minutes after the synapse was potentiated. LFS caused no depression of AMPAR-mediated transmission. Subsequent application of the AMPAR antagonist NBQX (10 μM) and depolarization of the postsynaptic cell to +40 mV revealed the NMDAR-only EPSC (right arrow), which was then compared with the NMDAR EPSC before synapse unsilencing (left arrow). Insets (1–5) show example traces at -65 mV (1) and +40 mV (2) prior to pairing; at -65 mV post-pairing (3), at -65 mV post-LFS (4) and at +40 mV during NBQX application (5). **B. **Data from six paired recording experiments, each performed as shown in part (A) showing that NMDAR-mediated transmission was unchanged by LFS in recently unsilenced synapses. Offset symbols show the average of the six experiments.

Because NMDAR responses could not be suppressed in silent synapses, it raises the possibility that like AMPARs [6], suppression of NMDA responses is dependent on the plastic state of the synapse. To test this idea, we attempted to depress NMDA responses in synapses that had been recently unsilenced. Our previous work has shown that AMPA receptor responses cannot be suppressed by LFS for the first 30 minutes following the awakening of a silent synapse, but that after 30 minutes has elapsed post-potentiation, AMPAR responses again become fully plastic [6]. We measured the amplitudes of NMDAR responses in silent synapses with the postsynaptic membrane potential held at +40 mV. These synapses were subsequently awakened by a standard LTP induction protocol, which resulted in an increase in AMPAR- but not NMDAR-mediated transmission [10]. Ten minutes after this potentiation, LFS was applied, which as previously reported did not suppress the AMPAR-mediated response [10]. Addition of NBQX and postsynaptic depolarization to +40 mV revealed the isolated NMDAR response, which could then be compared with the amplitudes of the NMDAR EPSCs before LFS (Figure 2A). LFS did not induce depression of the amplitude of this isolated NMDAR-mediated component in recently unsilenced synapses (Figure 2B; average NMDAR EPSC amplitudes were 10.3 ± 0.9 pA and 10.2 ± 0.8 pA (n = 6 pairs) before and after LFS respectively; p > 0.1). Nor did awakening of silent synapses change the decay time course of the NMDAR-mediated EPSC (Pre LFS: 79.5 ms +/- 10.8; post LFS: 82.0 ms +/- 4.6; difference not significant).

### NMDAR endocytosis at active synapses

**Figure 3:**
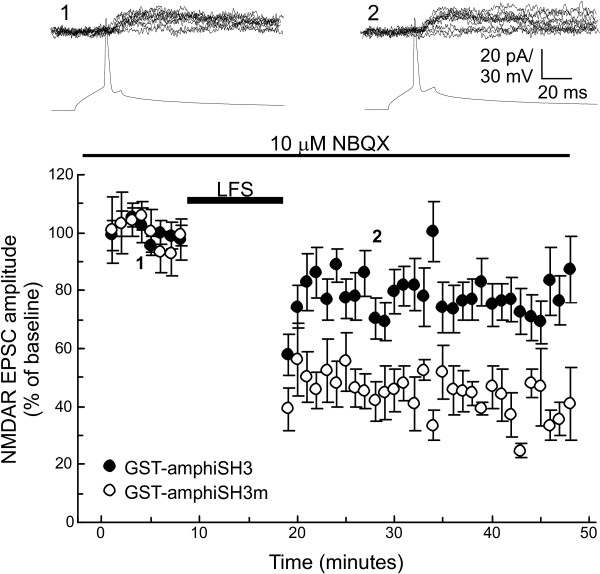
Prevention of dynamin-dependent endocytosis of NMDARs by injection of GST-amphiSH3 (100 μg/ml) into the postsynaptic cell significantly attenuates LFS-induced NMDAR down-regulation. The fusion protein was present in the postsynaptic internal solution for the duration of the experiment. Following the attainment of an active synaptic connection (characterized by the appearance of an AMPAR-mediated current at resting membrane potentials), NMDAR-mediated EPSCs were isolated by bath application of NBQX (10 μM) and depolarization to +40 mV. Baseline NMDAR EPSC amplitudes were measured for 10 minutes and then the synaptic pair was subjected to LFS (600 presynaptic action potentials at 1 Hz combined with slight postsynaptic depolarization; [6]). NMDAR EPSC amplitudes were subsequently measured for 30 minutes following LFS. Inset shows ten consecutive postsynaptic traces collected at +40 mV in NBQX, with an exemplar presynaptic action potential, before (1) and 10 minutes after (2) LFS. Closed symbols: GST-amphiSH3 (n = 10 pairs), open symbols: GST-amphiSH3m (n = 6 pairs) protein.

It has previously been shown that NMDAR currents at active synapses can be suppressed during synaptic depression. It is not known whether this LFS-induced down-regulation of NMDAR-mediated responses (Figure 1B) [6,18,19] is mediated by a decrease of receptor function, a decrease in presynaptic transmitter release or removal of the receptors from the membrane via endocytosis. While this decrease is known to be associated with a decline in the NMDA-sensitivity of the postsynaptic cell [6,19], this does not differentiate between different postsynaptic mechanisms. Recently, dynamin-dependent endocytosis of NMDARs has been shown to occur in acutely isolated neurons with exogenous glycine or glutamate application [23,24]. To determine whether dynamin-dependent endocytosis of NMDARs may occur following LFS at active synapses, we injected a glutathione S-transferase fusion protein of the amphiphysin SH3 domain (GST-amphiSH3) into the postsynaptic neurons of pyramidal cell pairs that were connected by an active synaptic connection. Previous work has shown that this protein binds to endogenous dynamin and thus competes with any endogenous interactions between amphiphysin and dynamin [13]. Basal amplitudes of synaptic transmission in active pairs were not significantly different whether the postsynaptic cell was injected with GST-amphiSH3 (100 μg/ml) or with the inactive mutant amphiSH3m [13]. The AMPAR-mediated current at -65 mV in GST-amphiSH3 and GST-amphiSH3m injected neurons were 20.3 ± 4.6 and 19.0 ± 6.0 respectively, (p >> 0.05). NMDAR currents were isolated by bath application of the AMPAR antagonist NBQX (10 μM; Figure 3) and their amplitudes measured by voltage clamping the postsynaptic cell at +40 mV. Ten minutes following the attainment of a stable NMDAR current baseline, LFS was applied. The presence of the dynamin interfering peptide (amphiSH3) in the postsynaptic cell significantly attenuated NMDAR LTD (Figure 3). Thirty minutes following LFS, average NMDAR current amplitudes were 87.1 ± 11.4% (n = 10 pairs) of pre-LFS baseline. NMDAR failure rates were not increased following LFS in the presence of GST-amphiSH3 (NMDAR failure rates before and after LFS were 24.4 ± 4.0 and 32.8 ± 4.0 respectively, p = 0.15). In contrast, postsynaptic injection of amphiSH3m (100 μg/ml) did not interfere with the expression of NMDAR LTD. Following LFS, NMDAR EPSC amplitudes measured 40.9 ± 12.4% of pre-LFS baseline; this was not significantly different from LTD induced in control experiments in which no peptide was present (average NMDAR EPSC amplitude was 42.5 ± 19.1% of baseline, 30 min following LFS, data from Figure 1B; n = 6 pairs, p >> 0.05), but was significantly different from NMDAR EPSC currents after LFS in the presence of GST-amphiSH3 (p < 0.02). In addition, in the presence of the mutant peptide, LFS resulted in a significant increase in NMDAR failure rates (20.4 ± 5.4 and 50.1 ± 7.0 before and after LFS respectively, p << 0.05).

## Discussion

The level of NMDAR activity in the postsynaptic membrane is a critical variable in defining the potential of a synapse to undergo plastic modifications. NMDAR-dependent regulation of AMPARs has been the dominant focus of research in recent years, with significant advances made in demonstrating the rapid changes in AMPAR expression via insertion and endocytosis into and out of the postsynaptic membrane. Given the dependence of many of these processes on the activation of the NMDAR, focus is beginning to turn to how activity can change NMDAR expression. We have shown that NMDARs are subject to activity-dependent down-regulation in a manner that depends on the recent history and plasticity state of the synapse. In silent synaptic connections, in which only functional NMDARs are present in the synaptic membrane, standard protocols that routinely depress both AMPAR- and NMDAR-mediated EPSCs at active synapses do not depress NMDAR-mediated currents. The fact that NMDAR responses cannot be depressed in silent synapses speaks not only to their dependence on synaptic state, but also reveals an important principal in the regulation of silent synapse number. Models of synapse elimination suggest that synapses can be removed as a result of competition between weaker and stronger synapses, with the stronger being maintained and the weaker removed. Our data suggest that the weakest synapses of all, silent synapses, are, in fact, stable in the face of low frequency stimulation. The protection of NMDARs at silent synapses from activity-dependent weakening would serve to stabilize this population of synapses, thus maintaining the maximum dynamic range available for increases in synaptic strength. The fact that both NMDAR- and AMPAR-mediated responses of active synapses can be suppressed by LFS suggests that it is they, not silent synapses that are more likely to be eliminated via a synaptic weakening mechanism.

At active synapses, LFS-induced changes in NMDAR-mediated transmission could occur via a number of possible mechanisms. We have shown here that this down-regulation is caused primarily by dynamin-mediated endocytosis of NMDARs. The accompanying increase in NMDAR failure rate is consistent with complete removal of NMDARs from a subset of the synapses between pyramidal cell pairs. The lack of change in NMDAR EPSC decay kinetics suggests that the state-dependent plasticity does not appear to be accompanied by a change in NMDAR subunit composition [25,26]. A small amount of depression of the NMDAR response remains after inhibition of the dynamin endocytic pathway. This likely represents an incomplete block of endocytosis by the injected protein, or alternatively that additional mechanisms such as use-dependent changes in receptor function [22], NMDARs diffusing to extrasynaptic sites [27] or changes in presynaptic transmitter release may make a small contribution to LTD. Earlier studies have suggested that such endocytosis of NMDARs does not occur with synaptic plasticity [14,15]. These studies employed different techniques, either pharmacological or field stimulation-induced alteration of synaptic strength in dissociated cultures, and measured changes in NMDAR expression using immunocytochemistry. These techniques may not be sensitive enough to detect activity-dependent changes in NMDAR expression measured by alternative methods [18,20-23]. In addition, a lack of change in NMDAR expression detected immunocytochemically could reflect the synaptic population being in a state in which NMDARs are not able to be internalized (e.g. recently silent).

Our results also demonstrate that while NMDA receptors can be endocytosed by synaptic activity, this appears to happen only when AMPA receptors are present in the synaptic membrane and are also available to be endocytosed. Specifically: 1) NMDARs cannot be endocytosed in silent synapses where AMPAR are absent from the membrane (Figure 1), 2) Neither NMDARs (Figure 2) nor AMPARs [6] are endocytosed in recently (<30 min) unsilenced synapses, but 3), both NMDAR and AMPAR responses are down-regulated in synapses unsilenced more than 30 minutes previously, and 4) NMDAR (Figure 3) and AMPAR [13] responses are both subject to LFS-induced dynamin-dependent endocytosis in active synapses. These observations are suggestive of a potential co-regulation of these two types of glutamate receptors c.f. [28], possibly through a physical coupling to the same endocytic machinery, or by one receptor subtype modifying an intrinsic property of the other thereby enabling its endocytosis.

## Conclusion

Independent of possible glutamate receptor co-regulation, it is clear that NMDARs are not static in the postsynaptic membrane, but are as dynamic as AMPARs with regard to synaptic depression. The mechanism of this NMDAR depression is endocytosis of the receptor molecules. The obvious conclusion is that synapses that have had their surface NMDARs reduced (or removed) are going to be less able to undergo future NMDAR-dependent changes in efficacy. Given the central role that NMDARs play in inducing synaptic plasticity, the observed state-dependent control of NMDAR expression shown in this study will provide a key regulatory role in determining the plastic potential of a synapse and could provide a key mechanism underlying metaplastic processes.

## Methods

### Whole-cell patch clamp

Hippocampal slices from P7-8 male rat pups were prepared [29,30] and maintained *in vitro *for 7–11 days before recording. Paired whole-cell recordings were performed by obtaining whole-cell recordings on two neighboring neurons in area CA3 [10,29]. Briefly, slices were immersed in recording artificial cerebrospinal fluid (ACSF) at room temperature, containing (in mM) 119 NaCl, 2.5 KCl, 1.3 MgSO_4_, 2.5 CaCl_2_, 1 Na_2_HPO_4_, 26.2 NaHCO_3_, 11 glucose, perfused at a rate of 2 mL/minute. Pyramidal cells in area CA3 were visualized by infrared DIC microscopy. Presynaptic neurons were held in standard current clamp mode using an Axoclamp 2A (Axon Instruments); unless otherwise stated, postsynaptic neurons were held in voltage clamp mode at -65 mV using an Axopatch 1C (Axon). Events were sampled at 10 kHz, and low-pass filtered 1–2 kHz. Each presynaptic action potential was induced by a 20 ms current pulse (typically 20–50 pA). Baseline EPSCs in response to presynaptic action potential firing were collected at 0.1 or 0.2 Hz. Internal solution consisted of (in mM): 120 K gluconate (presynaptic cell) or Cs gluconate (postsynaptic cell), 40 HEPES, 5 MgCl_2_, 0.3 NaGTP, 2 NaATP, 5 QX314 (postsynaptic cell only), pH 7.2 with KOH or CsOH.

### Synaptic plasticity induction and analysis

Long-term depression was induced by low-frequency presynaptic action potentials at 1 Hz paired with postsynaptic cell depolarization to ~-55 mV for 10 minutes. Potentiation was induced by pairing presynaptic action potentials at 1 Hz with postsynaptic depolarization to -10 to 0 mV (pairing) [6]. Depotentiation was attempted using the same induction protocol as LTD, and was performed 10 minutes following pairing-induced LTP. 'All-silent' synaptic connections were identified as previously described [10]. Silent synapses were awakened by the pairing protocol.

Series resistance (Rs) was continuously monitored throughout the duration of all recordings, and an experiment was discarded if Rs increased more than 20%. Synaptic failures were detected as described previously [10]. Synaptic excitatory postsynaptic current amplitudes graphed in figures represent average responses across all trials grouped in one minute bins and include failures. Possible changes in EPSC amplitude (both AMPAR and NMDAR-mediated) were measured immediately following the pairing or LFS protocols.

### NMDA receptor- mediated transmission

In some experiments performed on active synapses (i.e. having both AMPAR and NMDAR-mediated responses) it was necessary to view the NMDA response in isolation. This was accomplished by holding the postsynaptic membrane potential at +40 mV in the presence of the AMPAR antagonist NBQX (2,3-Dioxo-6-nitro-1,2,3,4-tetrahydrobenzo [f]quinoxaline-7-sulponamide disodium, 10 μM). NBQX was not required when recording NMDAR-mediated responses in silent synapses because they lack an AMPAR-mediated response, and therefore the NMDAR-mediated response is already isolated. Failures were defined as trials indistinguishable from pre-stimulus baseline and were detected as previously described [10]. 90–10% decay times were determined by software written in Labview by fitting a single exponential to synaptic NMDAR EPSCs [10].

### Silent synapses

When working with silent synaptic connections between two pyramidal neurons, the criteria used to identify a connection as "silent" are: 1) that it shows no AMPAR mediated postsynaptic current in response to the first 50 consecutive presynaptic action potentials in that recording, and, 2) that upon taking the postsynaptic membrane potential to positive values, an NMDAR-mediated EPSC be visible. As we have previously demonstrated, these are truly silent synapses, not synapses with a low probability of release [10]. It is also important to note that contrary to the misrepresentation of our data by Xiao, et al. [31]; these synapses are *found *silent, not created. This is known because, unlike what is written in Xiao, et al., we *did *use the initial AMPA response as control. The 50 consecutive AMPAR-responseless trials that formed part of our criterion were the *first *50 trials after the recording was obtained.

### Inhibition of endocytosis

To inhibit dynamin-dependent endocytosis the glutathione S-transferase fusion protein of the amphiphysin SH3 domain (GST-amphiSH3) [13,23] was added to the internal solution in the postsynaptic recording electrode at 100 μg/ml. Synaptically connected pairs were identified as being connected by active synapses, having AMPAR and NMDAR-mediated EPSCs. NMDAR-mediated currents were then isolated by application of NBQX. Baseline NMDAR-mediated transmission was monitored during the initial recording, while the peptide diffused into the postsynaptic cell. No change in baseline NMDA EPSC amplitude was recorded. The recording was maintained for a minimum of 15 minutes before LTD was induced to allow time for the peptide to diffuse into the postsynaptic cell. Identical experiments were performed as controls where an inactive mutant amphiphysin GST fusion protein (GST-amphiSH3m; 100 μg/ml) [13,23] was substituted for the active protein. The experimenter was blinded to which peptide was being used in each experiment.

### Data acquisition and analysis

Online data acquisition and offline analysis was performed with software written in Labview (Eric Schaible and Paul Pavlidis). Unless otherwise stated, all results are presented as mean ± standard error. Statistical significance was tested using the student t-test, with the level of significance set at p < 0.05.

## List of abbreviations

NMDA: N-methyl-D-aspartate

NMDAR: NMDA receptor

AMPA: α-amino-3-hydroxy-5-methylisoxazole-4-propionic acid

AMPAR: AMPA receptor

LTP: long-term potentiation

LTD: long-term depression

EPSP: Excitatory Postsynaptic Potential

EPSC: Excitatory Postsynaptic Current

LFS: Low Frequency Stimulus

GST-amphiSH3: glutathione S-transferase fusion protein of the amphiphysin SH3 domain

GST-amphiSH3m: mutant form of GST-amphiSH3, which is inactive

## Authors' contributions

JMM carried out almost all of the experiments and analysis illustrated in figures 1 and 2, along with some of the experiments in figure 3. JCS carried out most of the experiments and analysis illustrated in figure 3. JEH carried out experiments in support of these studies, and assisted in the data analysis and interpretation, DVM participated in the design and coordination of the study, along with data analysis and interpretation. The study was conceived by JMM and DVM. All authors read and approved the final manuscript.
